# Preoperative CYFRA 21-1 level as a prognostic indicator in resected primary squamous cell lung cancer.

**DOI:** 10.1038/bjc.1996.464

**Published:** 1996-09

**Authors:** J. Niklinski, M. Furman, T. Burzykowski, L. Chyczewski, J. Laudanski, E. Chyczewska, M. Rapellino

**Affiliations:** Department of Thoracic Surgery, Bialystok Medical Academy, Poland.

## Abstract

The CYFRA 21-1 assay is a test that has been developed recently for detection of a cytokeratin 19 fragment in serum. A diagnostic role for CYFRA 21-1 has already been proposed. The question of whether this marker is prognostically significant is important in clarifying the role of CYFRA 21-1 in clinical practice. The aim of this study was to evaluate the prognostic significance of elevated preoperative CYFRA 21-1 levels in patients with resected primary squamous-cell lung cancer (SqCC). Serum levels of CYFRA 21-1 were measured using an immunoradiometric assay (CIS bio) in 91 patients with operable SqCC. Survival and disease-free survival curves related to initial levels of this marker were estimated using the Kaplan-Meier method. In the univariate analysis the log-rank test and the log-rank test for trend were used. In the multivariate analysis the stratified log-rank test and the proportional hazard model were used. Elevated preoperative CYFRA 21-1 levels were identified in 55% of patients with SqCC. The number of patients with elevated levels of this marker increased with TNM stage (P = 0.0001). In univariate analysis elevated levels of CYFRA 21-1 were significantly associated with poor overall survival (P < 0.00005) and with disease-free survival (P < 0.00005). In multivariate analysis elevated levels of this marker were also found to be associated with poor overall and disease-free survival (P = 0.01 and P = 0.003 respectively). In conclusion, CYFRA 21-1 may be an independent prognostic parameter of survival and tumour relapse in SqCC and may be useful in identifying resected SqCC patients at high risk of treatment failure.


British Journal of Cancer (1996) 74, 956-960
© 1996 Stockton Press All rights reserved 0007-0920/96 $12.00

### Preoperative CYFRA 21-1 level as a prognostic indicator in resected primary squamous cell lung cancer

J Niklinski[1], M Furman[1], T Burzykowski[2], L Chyczewski[3], J Laudanski[1], E Chyczewska[4] and M Rapellino[5]

*[1]Department of Thoracic Surgery, Bialystok Medical Academy, Bialystok, Poland; [2]Biostatistical Unit, The Maria Sklodowska-Curie Memorial Cancer Center and Institute of Oncology, Warsaw, Poland; Departments of [3]Pathology and [4]Pneumonology, Bialystok Medical Academy, Bialystok, Poland; [5]Department of Pneumonology, University of Turin, Turin, Italy.*

**Summary** The CYFRA 21-1 assay is a test that has been developed recently for detection of a cytokeratin 19 fragment in serum. A diagnostic role for CYFRA 21-1 has already been proposed. The question of whether this marker is prognostically significant is important in clarifying the role of CYFRA 21-1 in clinical practice. The aim of this study was to evaluate the prognostic significance of elevated preoperative CYFRA 21-1 levels in patients with resected primary squamous-cell lung cancer (SqCC). Serum levels of CYFRA 21-1 were measured using an immunoradiometric assay (CIS bio) in 91 patients with operable SqCC. Survival and disease-free survival curves related to initial levels of this marker were estimated using the Kaplan-Meier method. In the univariate analysis the log-rank test and the log-rank test for trend were used. In the multivariate analysis the stratified log-rank test and the proportional hazard model were used. Elevated preoperative CYFRA 21-1 levels were identified in 55% of patients with SqCC. The number of patients with elevated levels of this marker increased with TNM stage ($P=0.0001$). In univariate analysis elevated levels of CYFRA 21-1 were significantly associated with poor overall survival ($P<0.00005$) and with disease-free survival ($P<0.00005$). In multivariate analysis elevated levels of this marker were also found to be associated with poor overall and disease-free survival ($P=0.01$ and $P=0.003$ respectively). In conclusion, CYFRA 21-1 may be an independent prognostic parameter of survival and tumour relapse in SqCC and may be useful in identifying resected SqCC patients at high risk of treatment failure.

**Keywords:** CYFRA 21-1; lung cancer; prognosis; tumour marker

Squamous-cell lung carcinoma comprises 40-50% of all cases of lung cancer. Its treatment is primarily surgical and its prognosis has remained almost unchanged over recent years. As is the case with other neoplasma, it would appear valuable to segregate patients with squamous cell lung carcinoma into different prognostic categories at the time of diagnosis because this approach may assist in the selection of types of treatment. Today, for patients with lung carcinoma, the TNM staging system is the most important prognostic factor. However, the variability of survival within staging groups required additional types of indicators, independent of stage to generate a 'comprehensive' estimate of prognosis (Fielding *et al.*, 1992).

Recently, great attention has been focused on the biology of lung cancer and a number of so-called tumour markers have been described (Richardson and Johnson, 1993; Buccheri and Ferigno, 1994; Mountain, 1995; Niklinski and Furman, 1995). One group of molecules that has been shown to be promising as tumour markers is the cytokeratins (Moll *et al.*, 1982).

Cytokeratins are one of the main families of intermediate filaments which make up the cytoskeleton. A cytokeratin is a heterotypic tetramer of protofilaments composed of two polypeptides: one acidic type I subunit and one basic type II subunit (Nagle, 1988). It is noteworthy that each type of epithelia and its malignant counterpart expresses a specific cytokeratin pattern. Immunohistochemical studies have demonstrated that simple epithelia, including respiratory epithelia, express cytokeratins 7, 8, 18 and 19 (Moll *et al.*, 1990). These cytokeratins, and particularly CK 19, are strongly expressed by lung cancer tissue (Broers *et al.*, 1988). Although cytokeratins are part of the cytoskeleton, some fragments might be released in the serum owing to cell lysis or tumour necrosis.

A new tumour marker assay (CYFRA 21-1) which uses two monoclonal antibodies (KS 19-1 and BM 19-21) against epitopes of a water-soluble fragment of CK 19, was recently introduced (Pujol *et al.*, 1993; Bodenmuler *et al.*, 1994).

The results of initial studies of CYFRA 21-1 in lung cancer patients showed its dominant value in squamous-cell carcinoma type (Pujol *et al.*, 1993; Stieber *et al.*, 1993; Bombardieri *et al.*, 1994; Rastel *et al.*, 1994; Rapellino *et al.*, 1995). The current study examines whether elevated serum CYFRA 21-1 levels have any prognostic value in patients with resected squamous-cell lung cancer.

### Material and methods

The study includes 91 squamous-cell lung cancer (SqCC) patients examined by the Chest Oncology Group and operated on in the Thoracic Surgery Unit at the Bialystok Medical School between May 1991 and June 1993. All of these patients underwent surgical resection. No patient underwent chemotherapy or radiation therapy before surgery.

Pretreatment staging procedures included physical and blood examinations, chest radiographs and tomographs, bronchoscopy, computed tomography (CT) of the thorax and ultrasound scanning of liver. In addition, radioisotopic scans of bones, examination of bone marrow aspirates and abdominal and brain CT scans were performed when necessary. Selected patients underwent mediastinoscopy. All patients were of good performance status at the time of surgery (Karnofsky, 80 to 100). During operation, radical lymph node dissection was uniformly performed. Nodes were identified and submitted separately at all levels. Pathological material has been specially reviewed for this study by the same pathologist. Post-operative, pathological staging (pTNM) (primary tumour, regional lymph involvement,

Correspondence and present address: J Niklinski, Laboratory of Biological Chemistry, National Cancer Institute, National Institutes of Health, Bldg. 37, R5D02, 9000 Rockville Pike, Bethesda MD 20852, USA
Received 23 January 1996; revised 27 March 1996; accepted 23 April 1996

occurrence of distant metastasis) was performed by correlating the operative and histological findings (Mountain, 1986). After surgery, patients were followed at 3 month intervals with a clinical and radiological examination.

At the time of our analysis, 30 patients had died. Two patients (2%) died of complications after surgery (operative death) and three patients died 33, 34 and 36 months after surgery, respectively, due to an unrelated cause. In these three cases, no tumour recurrence had been detected. A total of 25 remaining patients had died owing to recurrence of the disease. Three patients with recurrence are alive at 3 months after the second treatment (one patient was able to undergo a second resection, the other two were treated with radiation therapy).

In the whole group of patients (excluding the two patients who died of complications after surgery) the length of follow-up ranged from 11 to 38 months. In the group of 62 surviving patients, follow-up ranged from 11 to 36 months; 35 of those patients (57.4%) were observed for 36 months after surgery.

To determinate CYFRA 21-1 serum levels, venous blood samples were collected from each patient before surgery, centrifuged to obtain serum samples and stored at $-80°C$ until assayed. All samples were assayed in duplicate. Serum levels of CYFRA 21-1 were measured with immunoradiometric assay, using a commercial source (CIS bio international, Gif/Yvette, France), following the manufacturer's instructions. The cut-off point was 3.6 ng $ml^{-1}$.

*Statistical analysis*

Comparisons based on contingency tables were performed using the Fisher's exact test and the exact test for trend (Agresti, 1990). Survival time was calculated from the time of surgery to the date of death or the last observation of a patient. Patients alive at the end of the follow-up were regarded as censored observations. Disease-free survival time was calculated from the date of surgery to the date of the first occurrence of relapse or death or to the date of the last observation of a patient. Patients alive and free of disease at the end of the follow-up were regarded as censored observations.

Overall survival and disease-free survival curves related to initial serum CYFRA 21-1 level were estimated using the Kaplan–Meier method. Confidence intervals (95%) for the estimated probability of overall and disease-free survival were calculated using the asymptotic variance of the log-log transformation of the survivor function (Kalbfleisch and Prentice, 1980).

In the univariate analysis of the log-rank test (exact and asymptotic version) and the log-rank test for trend (asymptotic version; Tarone and Ware, 1977) were used. In the multivariate analysis the (exact) stratified log-rank test and proportional hazard model were used. Comparisons based on multivariate models were performed using the score test. The fit of models was checked both graphically and using time-dependent covariates (Kalbfleisch and Prentice, 1980).

All the tests performed in the univariate and the multivariate analyses were two-sided. The exact tests were computed using StatXact-Turbo v.2.0 software. Confidence intervals for the estimated probability of overall and disease-free survival were calculated using Stata v.3.0. The other methods were applied using BMDP/dynamic v.7.0 (programs 1l and 2l).

### Results

At the time of diagnosis 55% (50/91) of SqCC patients had CYFRA 21-1 levels higher than 3.6 ng $ml^{-1}$. Concentration of serum CYFRA 21-1 according to patients' characteristics is shown in Table I.

CYFRA 21-1 values differ significantly among groups defined by the TNM classification and age. When compared at 0.01 significance level (adjusted for the fact of performing multiple comparisons), it appears that the number of patients with elevated CYFRA 21-1 levels increases with TNM stage ($P=0.0001$), decreases with age ($P<0.00005$) and does not depend on sex (mid-$P=0.86$). Thus, there is a significant imbalance between the group of patients with a normal and an elevated CYFRA 21-1 level with respect to the distribution of TNM and age, which has to be taken into account in the analysis.

*Overall survival analysis*

In the analysis 91 patients were included. In this group of patients 30 deaths were observed. Four factors were investigated for their influence on the survival of the patients: sex, age (<50, 50–64, ⩾65 years), TNM stage (I, II, IIIa) and the level of CYFRA 21-1 (normal, ⩽3.6; elevated, >3.6 ng $ml^{-1}$).

The univariate analysis was based on the results of the log-rank test. Taking into account the fact that multiple comparisons were made, the 0.01 level of significance was adopted. The results of the test for age ($P<0.00005$), TNM ($P=0.005$) and CYFRA 21.1 level ($P<0.00005$) were found to be significant (Table II). The presence of an elevated level of CYFRA 21-1 was associated with a shorter survival time of patients (Figure 1).

In the multivariate analysis the (exact) stratified log-rank test for the level of CYFRA 21-1 factor, with strata defined by different combinations of age and TNM stage, was used first. The result ($P=0.02$) was significant at the 0.05 level (Table II). Secondly, an analysis using the proportional hazard model was performed. It was found that a stratified model (Table III), with two strata defined by TNM stage (I

**Table I** CYFRA21-1 serum levels categorised by patients' characteristics

| | *Mean* (*ng* $ml^{-1}$) | *Median* (*ng* $ml^{-1}$) | *Quartiles* *25% 75%* (*ng* $ml^{-1}$) | *No. of cases* (>*3.6 ng* $ml^{-1}$) | P-*value* |
|---|---|---|---|---|---|
| TNM | | | | | 0.0001[a] |
| I | 2.47 | 2.15 | 0.975 3.90 | 6/22 (27%) | |
| II | 3.75 | 3.40 | 2.05 5.20 | 18/37 (49%) | |
| IIIa | 9.10 | 9.70 | 5.90 11.875 | 26/32 (81%) | |
| Sex | | | | | 0.86[b] |
| M | 5.35 | 4.20 | 2.10 7.40 | 46/83 (55%) | |
| F | 5.06 | 3.75 | 2.175 9.15 | 4/8 (50%) | |
| Age | | | | | <0.00005[a] |
| <50 | 7.53 | 6.70 | 3.90 11.90 | 12/15 (80%) | |
| 50–64 | 6.38 | 5.40 | 3.55 7.675 | 34/46 (74%) | |
| >64 | 2.60 | 2.00 | 1.00 2.50 | 4/30 (13%) | |

[a] Exact test for trend; [b] mid *P*-value for the Fisher's exact test.

Table II Overall survival–the results of the univariate analysis

| | No. of deaths/ no. of patients | Estimated probability of surviving >2.5 years (with 95% CI) | P-value |
|---|---|---|---|
| CYFRA 21- | | | <0.00005[a] (0.02)[b] |
| ≤3.6 ng ml⁻¹ | 1/41 | 1.000[c] | |
| >3.6 ng ml⁻¹ | 29/50 | 0.501 (0.346–0.638) | |
| TNM | | | 0.005[d] |
| I | 4/22 | 0.889 (0.623–0.971) | |
| II | 8/37 | 0.811 (0.603–0.917) | |
| IIIa | 18/32 | 0.471 (0.281–0.640) | |
| Age (years) | | | <0.00005[d] |
| <50 | 10/15 | 0.429 (0.177–0.661) | |
| 50–64 | 19/46 | 0.593 (0.407–0.738) | |
| >64 | 1/30 | 1.000[c] | |
| Sex | | | 0.18[a] |
| M | 26/83 | 0.718 (0.592–0.810) | |
| F | 4/8 | 0.343 (0.117–0.814) | |

[a] Exact log-rank test; [b] exact stratified log-rank test (adjusted for the TNM and age); [c] confidence interval cannot be calculated; [d] log-rank test for trend based on the asymptotic distribution.

**Figure 1** Probability of survival of patients with normal and elevated preoperative CYFRA 21-1 level.

Table III Overall survival–proportional hazard model (stratified for TNM stage I and II *vs* IIIa)

| | RR | 95% CI for RR | P-value[a] |
|---|---|---|---|
| CYFRA 21-1 | | | |
| ≤3.6 ng ml⁻¹ | 1 | | |
| >3.6 ng ml⁻¹ | 15.91 | (1.51–167.2) | 0.01 |
| Age (years) | | | 0.48[b] (0.23[c]) |
| <50 | 1 | | |
| 50–64 | 0.66 | (0.28–1.56) | 0.34 |
| >64 | 0.32 | (0.03–3.51) | 0.34 |
| Sex | | | |
| F | 1 | | |
| M | 0.44 | (0.68–1.47) | 0.17 |

[a] For the score test; [b] overall *P*-value for the effect of age; [c] *P*-value for trend; the test was obtained by replacing by a linear covariate the two indicator variables for the age groups; the replacement does not introduce any qualitative changes to the conclusions based on the model presented in the table.

and II *vs* IIIa), gave the closest fit to the data. The score test for the null hypothesis that all the effects of the covariates included in the model are zero resulted in a *P*-value equal to 0.0001. The effect of an elevated CYFRA 21-1 level was found to be significant at the 0.05 significance level ($P=0.01$), with a relative risk estimate of 15.91.

### *Disease-free survival analysis*

In the group of 91 patients analysed, 28 recurrences and five deaths without recurrence were observed. Of 28 patients who relapsed, 25 died after a recurrence and three were alive after a second treatment. The same factors as in the overall survival analysis were considered.

In the univariate analysis (Table IV) the results of the log-rank tests for age ($P<0.00005$), TNM ($P=0.003$) and CYFRA 21-1 level ($P<0.00005$) were significant at the 0.01 significance level (adjusted for the multiple comparisons). The presence of an elevated level of CYFRA 21-1 was associated with a shorter disease-free survival time (Figure 2). The approach to the multivariate analysis was similar to the one chosen in the overall survival case. In the first step, the (exact) stratified log-rank test for CYFRA 21-1 level, with strata defined by different combinations of the age and TNM, was performed. It resulted in $P=0.003$, which was significant at the 0.05 significance level.

In an attempt to use the proportional hazard model it was found that there was a need to use a model stratified by age. However, fitting a model stratified by age with CYFRA 21-1 level as a covariate was impossible because the likelihood function appeared to be monotone in the coefficient related to the covariate (Bryson and Johnson, 1981). This was due to the fact that among younger patients (≤64 years) recurrences and deaths without recurrence were observed only for those having elevated levels of CYFRA 21-1, while among the oldest patients (>64 years) there were no recurrences and only one death was observed (for a patient with normal CYFRA 21-1 level). Since it was impossible to fit a model stratified by age, which would contain CYFRA 21-1 as a covariate, it was decided to limit the multivariate analysis of the disease-free survival to the use of the stratified log-rank test.

### Discussion

The conventional approach to assessing prognosis in patients with lung cancer has been to place great importance on stage of disease and anatomical and pathological variables, such as tumour size, histological subtype and degree of tumour differentiation. Recent advances in the understanding of lung tumour biology provide insights into many other potentially significant determinants of prognosis.

The possibility that long-term results or response to treatment are based on biological factors inherent in the tumour cells has been confirmed (Fielding *et al.*, 1992;

**Table IV** Disease-free survival–results of the univariate (log-rank test) and multivariate (stratified log-rank test) analysis

| | *No. of patients alive after relapse/No. of deaths after relapse* | *No. of patients alive without relapse/ No. of deaths without relapse* | *Estimated probability of surviving without relapse ⩾2.5 years (with 95% CI)* | P-*value* |
|---|---|---|---|---|
| CYFRA 21-1 | | | | <0.00005[a] (0.003)[b] |
| ⩽3.6 | 0/0 | 40/1 | 1.000[c] | |
| >3.6 | 3/25 | 18/4 | 0.455 (0.304–0.595) | |
| TNM | | | | 0.003[d] |
| I | 0/3 | 18/1 | 0.839 (0.579–0.945) | |
| II | 1/6 | 28/2 | 0.773 (0.561–0.891) | |
| IIIa | 2/16 | 12/2 | 0.463 (0.271–0.634) | |
| Age (years) | | | | <0.00005[d] |
| <50 | 2/9 | 3/1 | 0.429 (0.177–0.660) | |
| 50–64 | 1/16 | 26/3 | 0.541 (0.360–0.692) | |
| >64 | 0/0 | 29/1 | 1.000[c] | |
| Sex | | | | 0.13[a] |
| M | 3/21 | 54/5 | 0.698 (0.570–0.794) | |
| F | 0/4 | 4/0 | 0.365 (0.053–0.706) | |

[a] Exact log-rank test; [b] exact stratified log-rank test (adjusted for age and TNM); [c] confidence interval cannot be calculated; [d] log-rank test for trend based on the asymptotic distribution.

**Figure 2** Probability of disease-free survival of patients with normal and elevated preoperative CYFRA 21-1 level.

Niklinski and Furman, 1995). Additionally it has been suggested that the information that describes anatomical extent of tumour and additional types of prognostic marker should be combined

Considerable interest has been focused on a new biomarker-soluble fragment of cytokeratine 19 'CYFRA 21-1'. We now confirm that CYFRA 21-1 is a sensitive marker for SqCC and elevated levels of this marker were correlated with progression of tumour stage. In our previous study, we also found a significant relationship between the elevated levels of CYFRA 21-1 and mediastinal lymph node involvement (Niklinski *et al.*, 1994).

Since the diagnostic role of CYFRA 21-1 has already been presented (Stieber *et al.*, 1993; Ebert *et al.*, 1995; Paone *et al.*, 1995; Wieskopf *et al.*, 1995), it is worthwhile investigating whether pretreatment CYFRA 21-1 concentrations are related to the likelihood of relapse in lung cancer patients who undergo curative resection.

In this paper we report results aimed at the evaluation of CYFRA 21-1 as an independent prognostic factor to identify squamous-cell carcinoma patients at high risk of surgical treatment failure. We found that for the overall as well as disease-free survival the presence of an elevated level of CYFRA 21-1 was associated with a shorter failure time. For the overall and disease-free survival the negative effect of an elevated level of this marker was found to be statistically significant both in the univariate and multivariate analyses. Interestingly, in the group of 50 patients with an elevated CYFRA 21-1 level there were 29 deaths, while in the group of 41 patients with normal CYFRA 21-1 level only one death was observed. Among 50 patients with elevated CYFRA 21-1 level there were 28 recurrences, while in the group of 41 patients with a normal level of this marker no recurrences were observed. Additionally, among younger patients (⩽64 years) recurrences were observed only for those having elevated levels of CYFRA 21-1. These results suggest that the CYFRA 21-1 assay may be applicable to patients of a younger age group.

Others (Pujol *et al.*, 1993) found that CYFRA 21-1 was also an independent prognostic factor in their entire population of lung cancer (NSCLC and SCLC) along with performance status and disease stage.

Recently we reported the significant role of CYFRA 21-1 in post-operative monitoring of patients for the detection of tumour recurrence (Niklinski *et al.*, 1995). Curative surgery resulted in a significant drop of preoperatively elevated CYFRA 21-1 levels down to normal values, whereas steadily increasing marker concentrations predict or accompany clinical relapse.

Promising results have also been obtained in the comparison of the clinical value of CYFRA 21-1 assays for disease monitoring with WHO response criteria with therapy assessed using standard techniques (Gaast *et al.*, 1994). It was found that 84% of response evaluations yielded concordant results. More data, however, are needed to make sure whether the course of CYFRA 21-1 concentrations during therapy reflects precisely the disease status. The present study suggests that the determination of CYFRA 21-1 may provide additional, independent, prognostic information in squamous-cell lung cancer patients.

**Acknowledgements**

This study was supported by the Polish Scientific Programme (KBN 4 S403 007 07). The authors wish to thank the referees for their helpful suggestions.

### References

AGRESTI A. (1990). *Categorical Data Analysis*. 1st ed. Wiley: New York.

BODENMULLER H, OFENLOCH-HAHNLE B, LANE EB, DESSAUER A, BOTTGER V AND DONIE F. (1994). Lung cancer-associated keratin 19-fragments: development and biochemical characterization of the new seum Enzymum Test CYFRA 21-1. *Int. J. Biol. Markers*, **9**, 75–81.

BOMBARDIERI E, SEREGNI E, BOGNI A, ARDIT S, BELLOLI S, BUSETTO A, CANIELLO B, CASTELLI M, CIANETTI A, CORREALE M, DE ANGELIS G, GANDOLFO GM, GION M, MACCHIA V, MIONE R, NAVAGLIA F, ONETTO M, PAGANUZZI M, PECCHIO F, PLEBANI M, RAPELLINO M, RUGGERI G, VANNINI P, VITELLI G AND ZAMPERLIN A. (1994). Evaluation of cytokeratin 19 serum fragments (CYFRA 21-1) in patients with lung cancer: results of a multicenter trial. *Int. J. Biol. Markers*, **9**, 89–95.

BROERS JLV, RAMAEKERS FCS, ROT MK, OSTENDROP T, HUYSMANS A, VON MUIJEN GNP, WAGENAAR SS AND VOOJES GP. (1988). Cytokeratins in different types of human lung cancer as monitored by chain-specific monoclonal antibodies. *Cancer Res.*, **48**, 3221–3229.

BRYSON BC AND JOHNSON ME. (1981). The incidence of monotone likelihood in the Cox model. *Technometrics*, **23**, 381–383.

BUCCHERI G AND FERRIGNO D. (1994). Prognostic factors in lung cancer: tables and comments. *Eur. Respir. J.*, **7**, 1350–1364.

EBERT W, BODENMULLER H AND HOLZEL W. (1995). CYFRA 21-1–clinical applications and analytical requirements. *Scand. J. Clin. Lab. Invest.*, **55**, (suppl. 221), 72–80.

FIELDING LP, FENOGLIO-PREISER CM AND FREEDMAN LS. (1992). The future of prognostic factors in outcome prediction for patients with cancer. *Cancer*, **70**, 2367–2377.

KALBFLEISCH JD AND PRENTICE RL. (1980). *The Statistical Analysis of Failure Data*. Wiley: New York.

MOLL R, FRANKE WW, SCHILLER DL, GEIBZER B AND KREPLER R. (1982). The catalog of human cytokeratins: patterns of expression in normal epithelia, tumors and cultured cells. *Cell*, **31**, 11–24.

MOLL R, SCHILLER DL AND FRANKE WW. (1990). Identification of protein IT of the intestinal cytoskeleton as a novel type I cytokeratin with unusual properties and expression patterns. *J. Cell Biol.*, **111**, 567–580.

MOUNTAIN CF. (1986). A new international staging system for lung cancer. *Chest*, **89**, (suppl.), 225–233.

MOUNTAIN CF. (1995). New prognostic factors in lung cancer: biologic prophets of cancer cell aggression. *Chest*, **108**, 246–254.

NAGLE R. (1988). Intermediate filaments: a review of the basic biology. *Am. J. Surg. Pathol.*, **12** (suppl.), 4–16.

NIKLINSKI J AND FURMAN M. (1995). Clinical tumour markers in lung cancer. *Eur. J. Cancer Prev.*, **4**, 129–138.

NIKLINSKI J, FURMAN M, CHYCZEWSKA E, CHYCZEWSKI L, ROGOWSKI F, JAROSZEWICZ E AND LAUDANSKI J. (1994). Evaluation of CYFRA 21-1 as a new marker for non-small cell lung cancer. *Eur. J. Cancer Prev.*, **3**, 227–230.

NIKLINSKI J, FURMAN M, CHYCZEWSKA E, CHYCZEWSKI L, ROGOWSKI F AND LAUDANSKI J. (1995). Diagnostic and prognostic value of the new tumour marker CYFRA 21-1 in patients with squamous cell lung cancer. *Eur. Respir. J.*, **8**, 291–294.

PAONE G, DE ANGELIS G, MUNNO R, PALLOTTA G, BIGIONI D, SALTINI C, BISETTI A AND AMEGLIO F. (1995). Discriminant analysis on small cell lung cancer and non-small cell lung cancer by means of NSE and CYFRA-21.1. *Eur. Respir, J.*, **8**, 1136–1140.

PUJOL JL, GRENIER J, DAURES JP, DAVER A, PUJOL H AND MICHEL FB. (1993). Serum fragment of cytokeratin subunit 19 measured by CYFRA 21-1 immunoradiometric assay as a marker of lung cancer. *Cancer Res.*, **53**, 61–66.

RAPELLINO M, NIKLINSKI J, PECCHIO F, FURMAN M, BALDI S, CHYCZEWSKI L, RUFFINI E AND CHYCZEWSKA E. (1995). CYFRA 21-1 as a tumour marker for bronchogenic carcinoma. *Eur. Respir. J.*, **8**, 407–410.

RASTEL D, RAMAIOLI A, CORNILLIE F AND THIRION B. (1994). CYFRA 21-1 a sensitive and specific new tumour marker for squamous cell lung cancer. Report of the first European multicenter evaluation. CYFRA 21-1 Multicentre Study Group. *Eur. J. Cancer*, **30A**, 601–605.

RICHARDSON GE AND JOHNSON BE. (1993). The biology of lung cancer. *Semin. Oncol.*, **20**, 105–127.

STIEBER P, HASHOLZNER U, BODENMULLER H, NAGEL D, SUNDER-PLASSMANN L, DIENEMANN H, MEIER W AND FATEH-MOGHADAM A. (1993). CYFRA 21-1: A new marker in lung cancer. *Cancer*, **72**, 707–713.

TARONE RE AND WARE J. (1977). On distribution-free tests for equality of survival distributions. *Biometrika*, **64**, 156–160.

VAN DER GAAST A, SCHOENMAKERS CHH, KOK TC, BLIJENBERG BG, CORNILLE F AND SPLINTER TAW. (1994). Evaluation of a new tumour marker in patients with non-small-cell lung cancer: CYFRA 21-1. *Br. J. Cancer*, **69**, 525–528.

WIESKOPF B, DEMANGEAT C, PUROHIT A, STENGER R, GRIES P, KREISMAN H AND QUOIX E. (1995). Cyfra 21-1 as a biologic marker of non-small cell lung cancer. Evaluation of sensitivity, specificity, and prognostic role. *Chest*, **108**, 163–169.


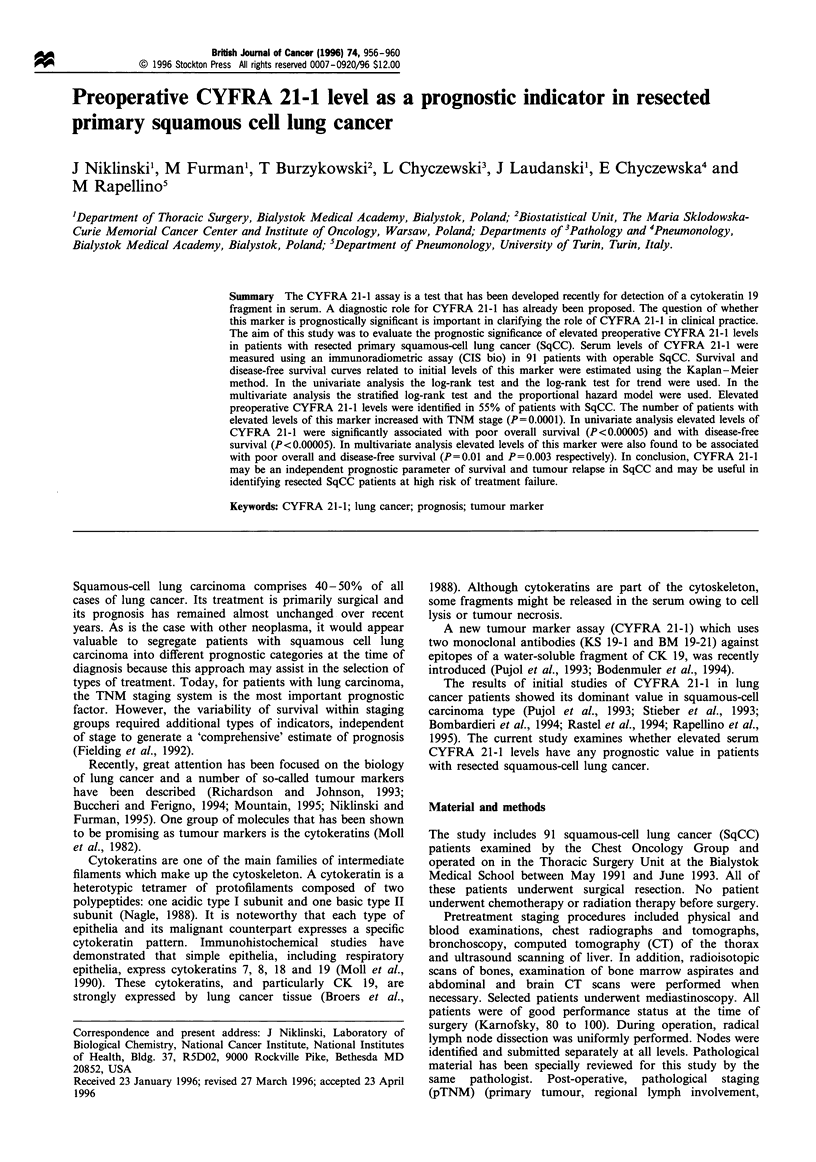

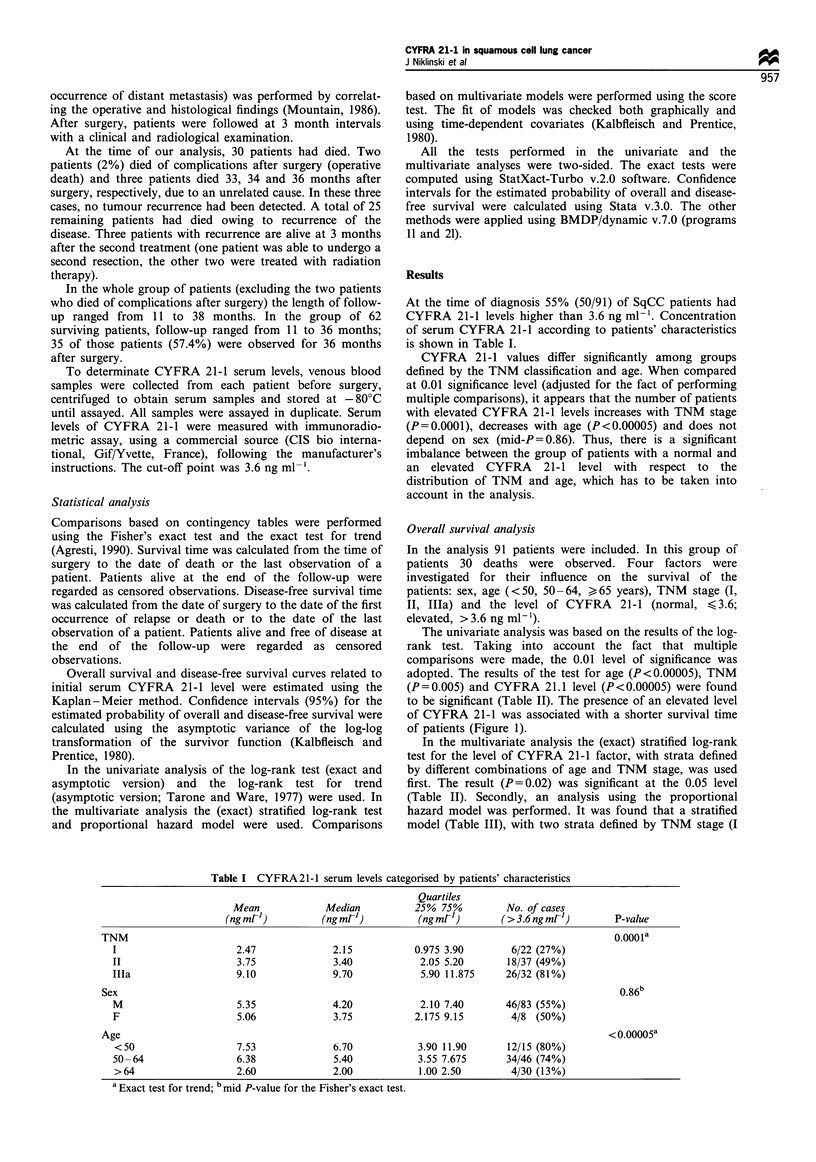

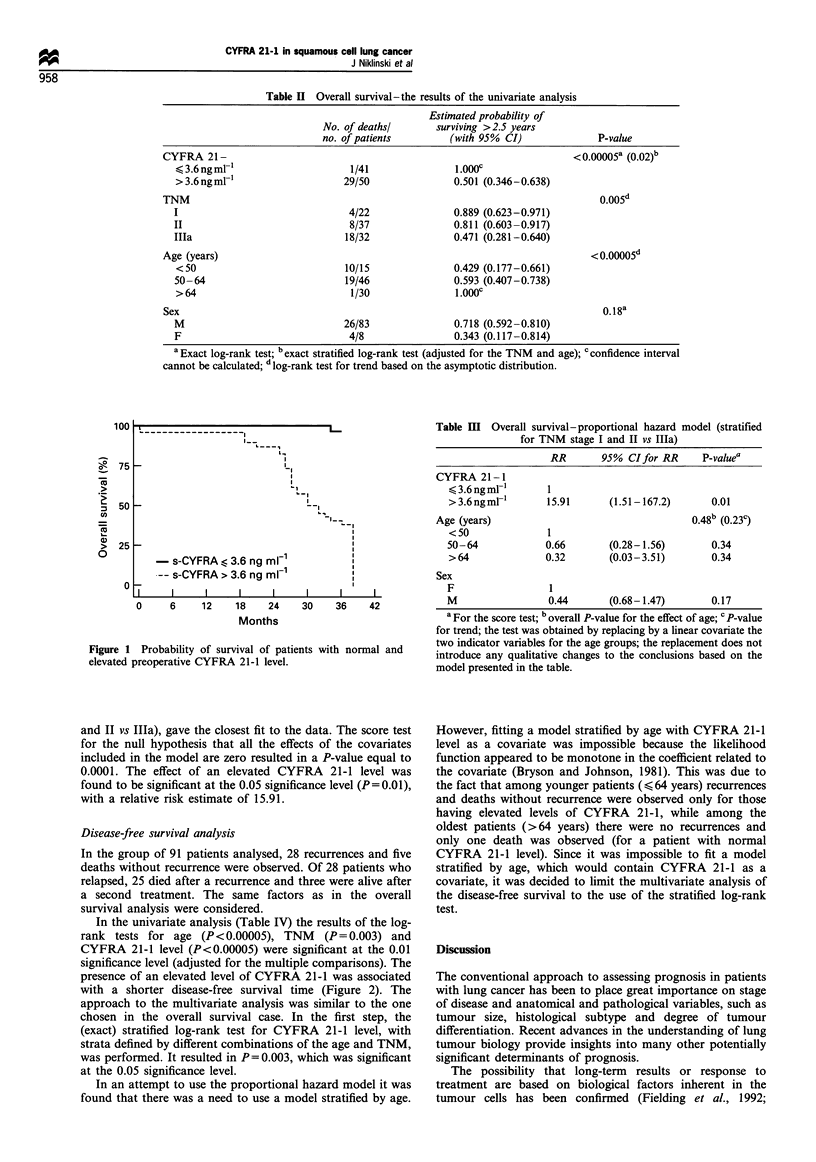

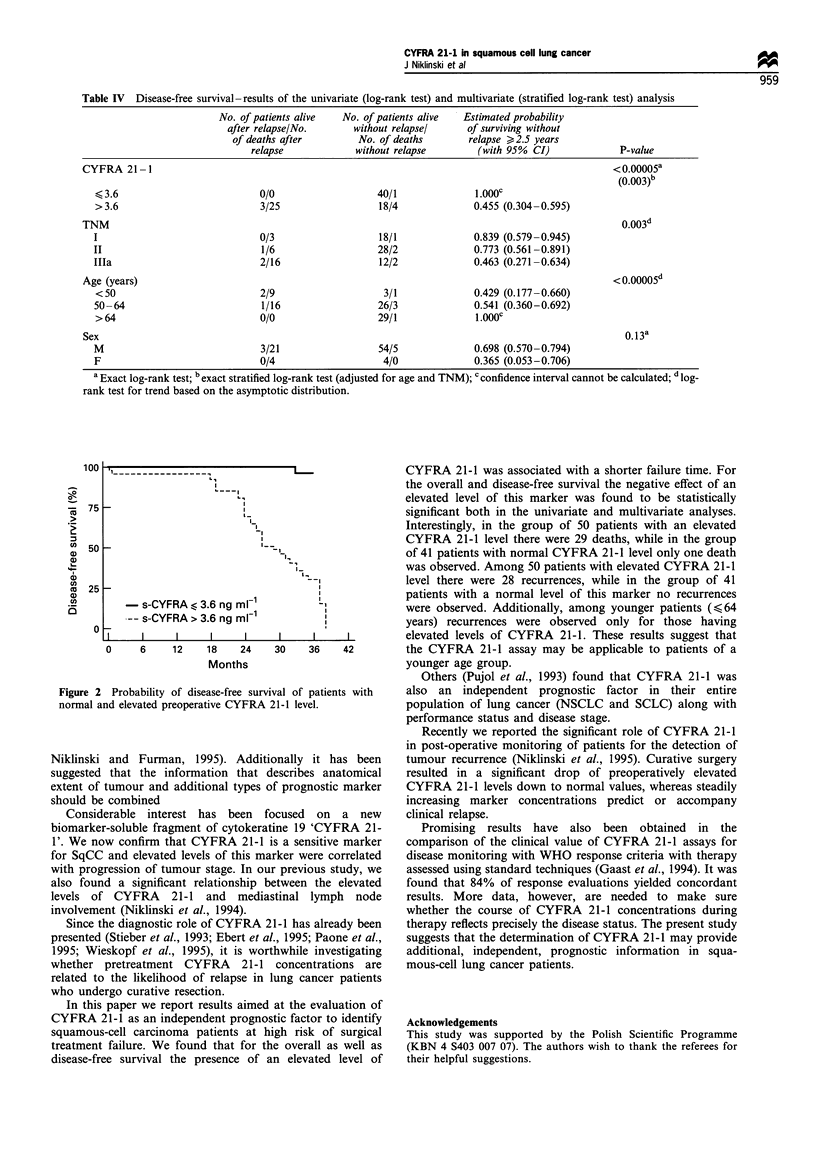

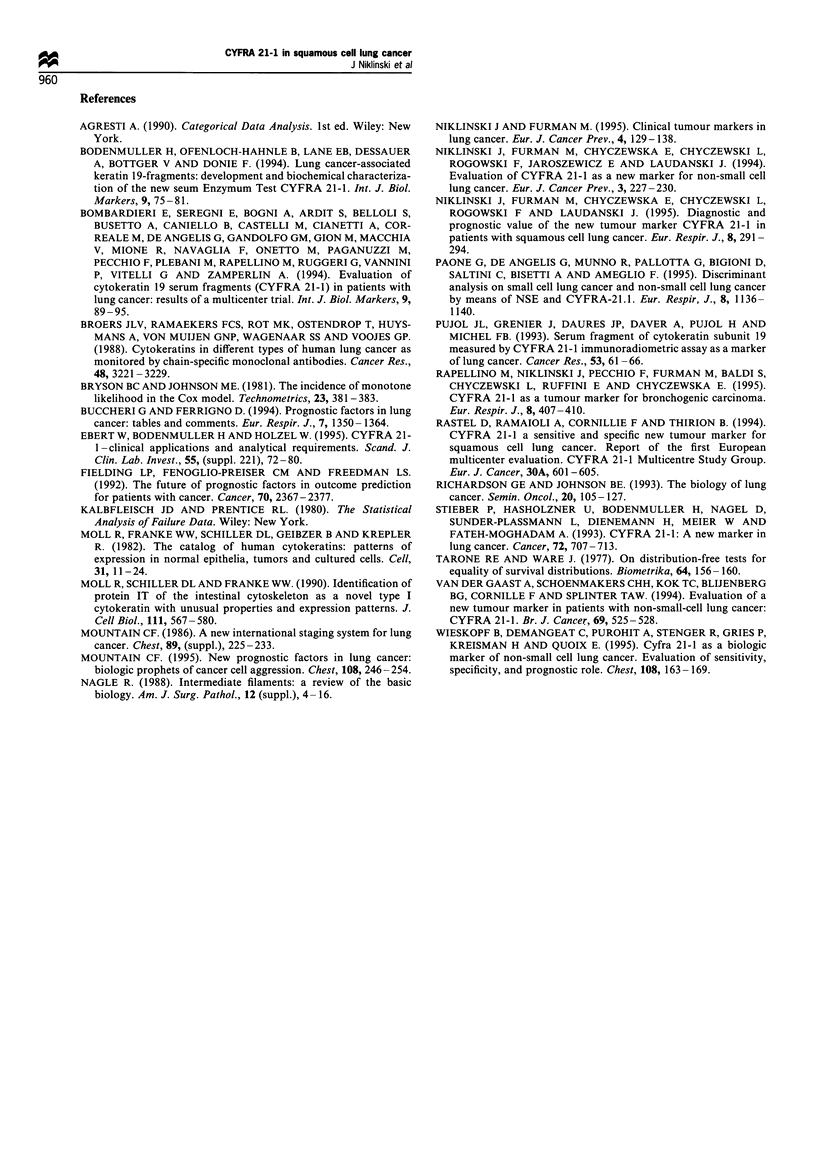


## References

[OCR_00585] Bodenmüller H., Ofenloch-Hähnle B., Lane E. B., Dessauer A., Böttger V., Donié F. (1994). Lung cancer-associated keratin 19 fragments: development and biochemical characterisation of the new serum assay Enzymun-Test CYFRA 21-1.. Int J Biol Markers.

[OCR_00604] Broers J. L., Ramaekers F. C., Rot M. K., Oostendorp T., Huysmans A., van Muijen G. N., Wagenaar S. S., Vooijs G. P. (1988). Cytokeratins in different types of human lung cancer as monitored by chain-specific monoclonal antibodies.. Cancer Res.

[OCR_00612] Buccheri G., Ferrigno D. (1994). Prognostic factors in lung cancer: tables and comments.. Eur Respir J.

[OCR_00616] Ebert W., Bodenmüller H., Hölzel W. (1995). CYFRA 21-1--clinical applications and analytical requirements.. Scand J Clin Lab Invest Suppl.

[OCR_00623] Fielding L. P., Fenoglio-Preiser C. M., Freedman L. S. (1992). The future of prognostic factors in outcome prediction for patients with cancer.. Cancer.

[OCR_00630] Moll R., Franke W. W., Schiller D. L., Geiger B., Krepler R. (1982). The catalog of human cytokeratins: patterns of expression in normal epithelia, tumors and cultured cells.. Cell.

[OCR_00638] Moll R., Schiller D. L., Franke W. W. (1990). Identification of protein IT of the intestinal cytoskeleton as a novel type I cytokeratin with unusual properties and expression patterns.. J Cell Biol.

[OCR_00644] Mountain C. F. (1995). New prognostic factors in lung cancer. Biologic prophets of cancer cell aggression.. Chest.

[OCR_00649] Nagle R. B. (1988). Intermediate filaments: a review of the basic biology.. Am J Surg Pathol.

[OCR_00660] Niklinski J., Furman M., Chyczewska E., Chyczewski L., Rogowski F., Jaroszewicz E., Laudanski J. (1994). Evaluation of CYFRA 21-1 as a new marker for non-small cell lung cancer.. Eur J Cancer Prev.

[OCR_00663] Niklinski J., Furman M., Chyczewska E., Chyczewski L., Rogowski F., Laudanski J. (1995). Diagnostic and prognostic value of the new tumour marker CYFRA 21-1 in patients with squamous cell lung cancer.. Eur Respir J.

[OCR_00655] Niklinski J., Furman M. (1995). Clinical tumour markers in lung cancer.. Eur J Cancer Prev.

[OCR_00673] Paone G., De Angelis G., Munno R., Pallotta G., Bigioni D., Saltini C., Bisetti A., Ameglio F. (1995). Discriminant analysis on small cell lung cancer and non-small cell lung cancer by means of NSE and CYFRA-21.1.. Eur Respir J.

[OCR_00680] Pujol J. L., Grenier J., Daurès J. P., Daver A., Pujol H., Michel F. B. (1993). Serum fragment of cytokeratin subunit 19 measured by CYFRA 21-1 immunoradiometric assay as a marker of lung cancer.. Cancer Res.

[OCR_00686] Rapellino M., Niklinski J., Pecchio F., Furman M., Baldi S., Chyczewski L., Ruffini E., Chyczewska E. (1995). CYFRA 21-1 as a tumour marker for bronchogenic carcinoma.. Eur Respir J.

[OCR_00691] Rastel D., Ramaioli A., Cornillie F., Thirion B. (1994). CYFRA 21-1, a sensitive and specific new tumour marker for squamous cell lung cancer. Report of the first European multicentre evaluation. CYFRA 21-1 Multicentre Study Group.. Eur J Cancer.

[OCR_00696] Richardson G. E., Johnson B. E. (1993). The biology of lung cancer.. Semin Oncol.

[OCR_00700] Stieber P., Hasholzner U., Bodenmüller H., Nagel D., Sunder-Plassmann L., Dienemann H., Meier W., Fateh-Moghadam A. (1993). CYFRA 21-1. A new marker in lung cancer.. Cancer.

[OCR_00716] Wieskopf B., Demangeat C., Purohit A., Stenger R., Gries P., Kreisman H., Quoix E. (1995). Cyfra 21-1 as a biologic marker of non-small cell lung cancer. Evaluation of sensitivity, specificity, and prognostic role.. Chest.

[OCR_00710] van der Gaast A., Schoenmakers C. H., Kok T. C., Blijenberg B. G., Cornillie F., Splinter T. A. (1994). Evaluation of a new tumour marker in patients with non-small-cell lung cancer: Cyfra 21.1.. Br J Cancer.

